# A Comparative Study of 2-Corner, 3-Corner, and 4-Corner Arthrodesis for Midcarpal Arthritis

**DOI:** 10.1177/15589447231174046

**Published:** 2023-06-02

**Authors:** Justine Ring, Tod A. Clark, Jennifer L. Giuffre

**Affiliations:** 1 University of Manitoba, Winnipeg, Canada; 2 Pan Am Clinic, Winnipeg, Manitoba, Canada

**Keywords:** arthritis, patient outcome assessment, bones, carpal, bones, scaphoid, arthrodesis

## Abstract

**Background:**

Four-corner fusion (4CF) is a common treatment for midcarpal arthritis; however, alternatives including 2-corner fusion (2CF) and 3-corner fusion (3CF) have been described. Limited literature suggests 2CF and 3CF may improve range of motion but have higher complication rates. Our objective is to compare function and patient-reported outcomes following 4CF, 3CF, and 2CF at our institution.

**Methods:**

Adult patients undergoing 4CF, 3CF, and 2CF from 2011 to 2021 who attended at least one follow-up were included. Four-corner fusion patients were compared with those who underwent either 3CF or 2CF using staple fixation. Outcomes include nonunion rate, reoperation rate, progression to wrist fusion, range of motion, and patient-reported pain, satisfaction, and Disabilities of the Arm, Shoulder, and Hand (DASH) scores.

**Results:**

A total of 58 patients met inclusion criteria. There were 49 4CF and 9 2CF or 3CF patients. Nonunion rates, progression to wrist fusion, and repeat surgery for any indication were not significantly different among groups. Range of motion (flexion-extension, radial-ulnar deviation) and grip strength at postoperative visits were not significantly different. Significantly more 4CF patients required bone grafting. Pain, overall satisfaction, and DASH scores were similar.

**Conclusions:**

Although prior studies suggest increased risk of nonunion and hardware migration after 2CF/3CF, we did not observe higher complication rates compared with 4CF. Range of motion, strength, and patient-reported outcomes were similar. While 4CF is traditionally the procedure of choice for midcarpal fusion, we found that when using a staple fixation technique, 2CF and 3CF have comparable clinical and patient-reported outcomes yet decrease the need for autologous bone grafting.

## Introduction

Traditionally, a proximal-row carpectomy (PRC) and a 4-corner fusion (4CF)^1^ are motion-sparing alternatives to wrist arthrodesis in high-functioning patients with advanced scapholunate advanced collapse (SLAC) and scaphoid nonunion advanced collapse (SNAC) wrists. Several studies and 2 systematic reviews comparing PRC and 4CF have demonstrated both procedures to be effective in decreasing pain and improving grip strength and range of motion,^2,3^ thereby making the choice of procedure patient and surgeon dependent.

In 1984, Watson and Ballet ^4^ initially described the 4CF as a treatment for SLAC wrist; however, they also described a limited arthrodesis between the capitate and lunate (2-corner fusion [2CF]). The proposed advantages of a 2CF are shorter operating times, less need for bone grafting, and greater flexion-extension arcs. Excision of the triquetrum is occasionally performed concomitant with the 2CF to prevent the complication of pisotriquetral arthritis. The 2CF was initially associated with higher nonunion rates,^5^ leading to less widespread adoption than 4CF. Recently, reports of 2CFs using compression screws rather than k-wires have demonstrated comparable nonunion rates with those reported for 4CF;^6,7^ although, screw migration remains problematic.^8^ The alternative 3-corner fusion (3CF) involves limited arthrodesis between the lunocapitate and capitohamate joints and has similar advantages to 2CF.^9,10^

Studies comparing various midcarpal arthrodesis procedures remain limited. Gauci et al^11^ demonstrated similar rates of nonunion, progression to total wrist arthrodesis (TWA), and range of motion in a retrospective cohort of SNAC and SLAC wrists who underwent 3CF or bicolumnar arthrodesis (BA) of the lunocapitate and triquetral-hamate joints. In the only comparative study, Gaston et al^12^ retrospectively compared outcomes following 2CF and 4CF in 57 patients with a diagnosis of SNAC wrist. They found no difference in progression to TWA, nonunion, or patient-reported outcomes, and reported a slight increase in flexion-extension arc after 2CF. Gaston et al^12^ also reported a 32% rate of screw migration requiring removal in the 2CF group, despite rates of nonunion being comparable to 4CF. While Gaston^13^ later reported nonunion rates of 0%, previous case series of 2CF reported nonunion rates between 6% and 14%.^5,6^ More recent case series have reported reduced rates of hardware complications in 2CF using a staple fixation technique rather than screw fixation.^13^ To date, there are no studies comparing nonunion rates, conversion to TWA, and wrist range of motion when staple fixation is used between the traditional 4CF and 2CF and 3CF.

The objective of this study is to compare 4CF to 3CF and 2CF at our institution. We aimed to identify differences in overall rates of revision surgery, progression to TWA, and nonunion between techniques. Secondarily, we aim to compare functional outcomes including wrist range of motion and grip strength, as well as patient-reported pain, satisfaction, and Disabilities of the Arm, Shoulder, and Hand (DASH) scores.

## Materials and Methods

A single-center cohort study was conducted at the University of Manitoba of all adult patients who underwent limited midcarpal arthrodesis from 2011 to 2021. All procedures followed were in accordance with the ethical standards of the responsible committee on human experimentation (institutional and national) and with the Helsinki Declaration of 1975, as revised in 2008(5). Informed consent was obtained from all individual participants included in the study. Research ethics were approved by the University of Manitoba Bannatyne Research Ethics Board. Clinical and functional outcomes were assessed through retrospective chart review, and patient-reported outcome measures through a well-validated visual-analog scale (VAS) for current pain, and Quick Disabilities of the Arm, Shoulder, and Hand (Quick DASH). Post-operation, all patients were treated with a minimum of 6 weeks of casting and referred to physiotherapy for increasing active and passive range of motion, with the frequency and duration of the program at the discretion of the treating therapist. All patients included in the study attended a minimum of one postoperative appointment following 6 weeks of casting.

## Inclusion Criteria

All patients who underwent a midcarpal arthrodesis, including 4CF, 3CF, and 2CF by either of 2 fellowship-trained hand surgeons at our institution were included. All patients were adults (>18 years of age) at the time of their initial surgery.

## Exclusion Criteria

Patients who did not attend at least one follow-up appointment following 6 weeks of casting were excluded.

## Expected Data Elements

Demographic information including age at initial surgery, sex, handedness, smoking status, prior operations on the involved hand, diabetes, months of follow-up, and preoperative diagnosis was collected for all patients. The type of midcarpal arthrodesis (2CF, 3CF, or 4CF) was recorded, as well as the method of fixation used (staples, k-wires, or compression screws). The need for bone autografting and the donor site of the bone graft were recorded for all patients. Any additional surgical procedures undertaken at the time of midcarpal fusion were recorded.

Primary objective outcomes include conversion to TWA, repeat midcarpal arthrodesis, rates of nonunion, symptomatic hardware requiring removal, and chronic postoperative pain requiring either additional surgery or referral to a pain specialist. Further surgery to the involved hand for any indication was recorded. In addition, total flexion and extension arc (°), radial and ulnar deviation arc (°), pronation and supination (°), and grip strength (kg) were measured.

Three patient-reported outcomes are included. First, patients are asked if overall, they are satisfied or unsatisfied with their initial surgery (4CF, 3CF, or 2CF). Second, the VAS for current pain, with a rating from 0 (no pain) to 10 (worst pain), is collected. Third, the Quick DASH is administered to all patients, including (optional) work and sports/performing arts modules when applicable.

## Statistical Analysis

Statistical analysis was conducted using SPSS Version 27. Demographics and all outcome data elements were compared between 4CF and limited partial arthrodesis techniques (2CF, 3CF). Normality testing was conducted for all data elements using the Shapiro-Wilk test. Categorical variables were compared using Fisher’s exact test and continuous variables were compared using the 2-tailed *t* test when the data were normally distributed. When the data were not normally distributed, the chi-squared and Mann-Whitney *U* tests were used for categorical and continuous variables, respectively. Statistical significance was set to *P* < .05 with a confidence interval of 95%.

## Results

**Figure 1.**
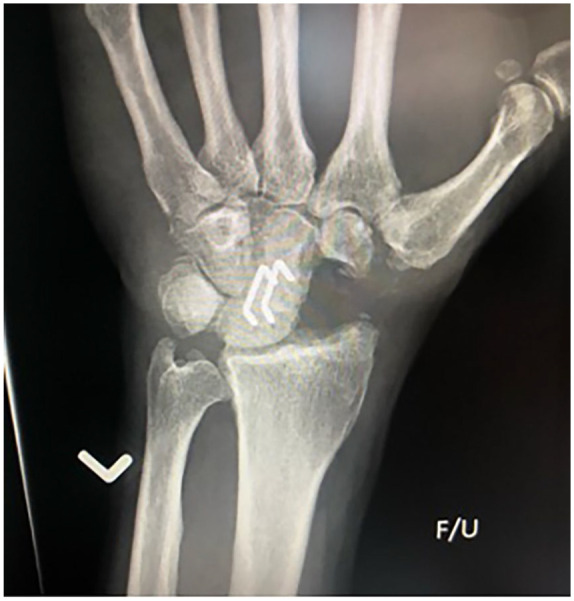
Anteroposterior radiograph of a 2-corner fusion using staples between the lunate and capitate. The scaphoid has been excised.

**Figure 2.**
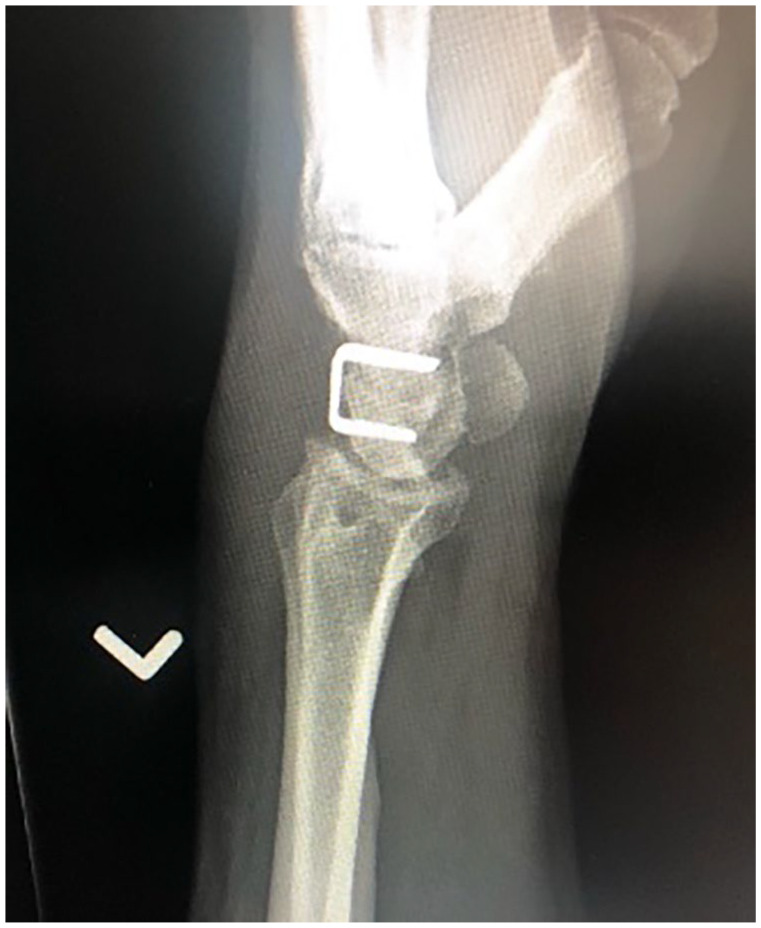
Lateral radiograph of a 2-corner fusion using staples between the lunate and capitate. The scaphoid has been excised.

**Figure 3.**
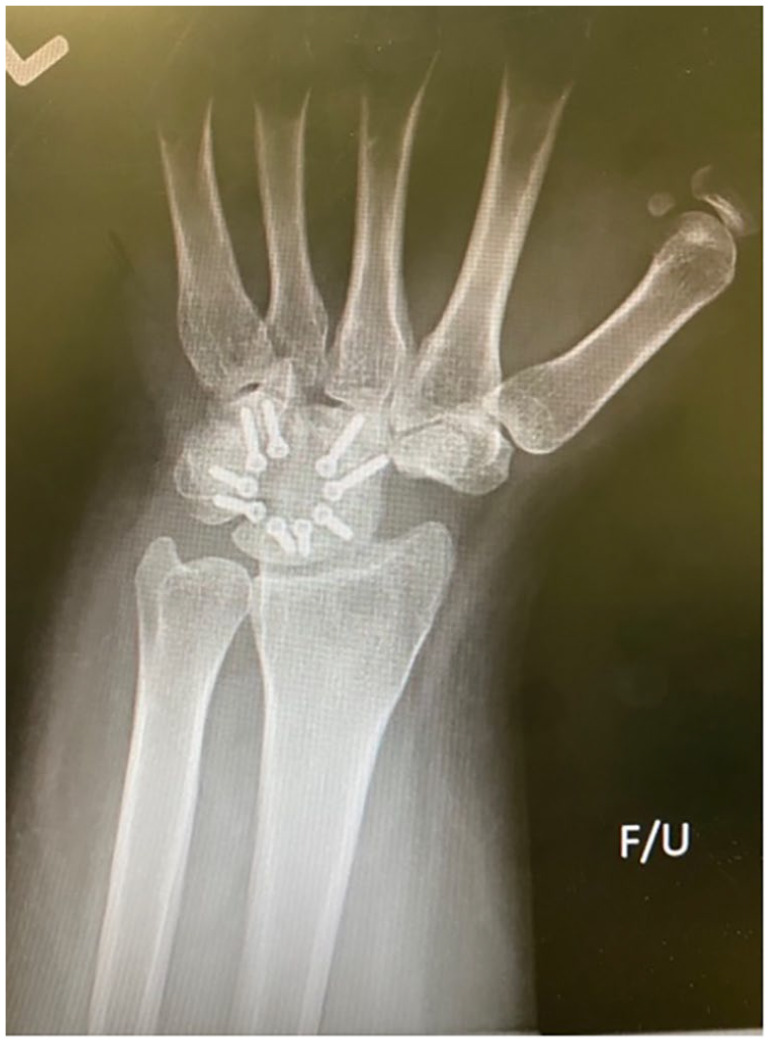
Anteroposterior radiograph of a 4-corner fusion using a circular plate for fixation of the capitate, hamate, lunate, and triquetrum. The scaphoid has been excised.

**Figure 4.**
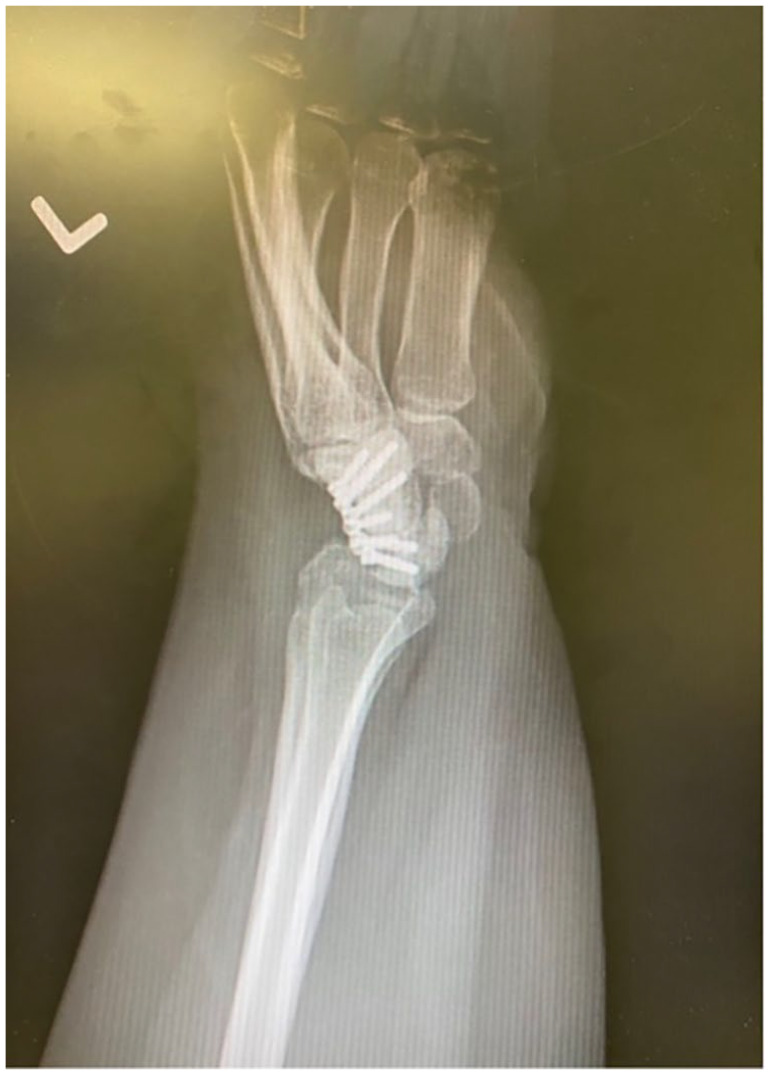
Lateral radiograph of a 4-corner fusion using a circular plate for fixation of the capitate, hamate, lunate, and triquetrum. The scaphoid has been excised.

A total of 48 patients undergoing 58 procedures met the inclusion criteria: 49 4CF (84%), 8 2CF (14%), and 1 3CF (2%). The 2CF and 3CF were grouped together and compared with 4CF (Table 1). All 2CF and 3CF were achieved using 2 staples between the lunate and capitate (2CF; Figures 1 and 2) and a third between the capitate and hamate (3CF). Of the 4CF procedures, 24 used staples and 25 used round plates (Figures 3 and 4).

**Table 1.** Baseline Demographics of Patients Meeting Inclusion Criteria (n = 58).

Data	4CF (n = 49)	2CF and 3CF (n = 9)	*P* value
Age, mean, y	55.4 ± 12.8	53.1 ± 13.1	.341
Sex	38 M 11 F	7 M 2 F	.988
Follow-up, mean, mo	10.4 ± 11.1	9.1 ± 15.1	.826
Smoker (current)	7 (14.3%)	1 (11.1%)	.800
Diabetes	2 (4.1%)	0 (0%)	.537
Diagnosis			
SLAC wrist	35 (71.4%)	6 (66.7%)	.956
SNAC wrist	9 (18.4%)	2 (22.2%)	
Other	5 (10.2%)	1 (11.1%)	
Prior hand/wrist surgery	13 (26.5%)	2 (22.2%)	.786
Bone grafting	42 (85.7%)	2 (22.2%)*	<.001

*Note.* 4CF = 4-corner fusion; 2CF = 2-corner fusion; 3CF = 3-corner fusion; SLAC = scapholunate advanced collapse; SNAC = scaphoid nonunion advanced collapse; M = male; F = female.

* *p*<0.05.

Patient age ranged from 18 to 72 years (45 (78%) males; 13 (22%) females; Table 1). Average follow-up was 10.7 (range, 2-49) months. Eight patients (14%) were active smokers at surgery. Of 58 wrists, 11 (19%) had a preoperative diagnoses of SNAC wrist and 41 (71%) SLAC wrist. Six (10%) had “other” diagnosis, including perilunate injuries (n = 2, 3%) and prior midcarpal nonunion (n = 1, 2%).

### Clinical Outcomes

Bone grafting was used significantly more in 4CF (n = 42, 86%) than in 2CF/3CF (n = 2, 22%; *P* < .001; Table 1), from the scaphoid (n = 13, 30%) or distal radius (n = 31, 70%).

Nine patients underwent additional surgery: 8 4CF patients and 1 2CF/3CF patient (*P* = .691; Table 2). Of the 4CF patients, 3 required total wrist fusion, 1 required hardware removal and a Sauve-Kapandji procedure, and 4 underwent revision fusion. The 2CF/3CF patient underwent conversion to 4CF.

**Table 2.** Clinical and Functional Outcomes, Recorded a Minimum of 6 Weeks After 4CF or 2CF/3CF.

Data	4CF (n = 49)	2CF and 3CF (n = 9)	*P* value
Revision surgery (any)	8 (16.3%)	1 (11.1%)	.691
Progression to wrist fusion	3 (6.1%)	0 (0%)	.597
Nonunion	5 (10.2%)	1 (11.1%)	.935
Flexion-extension arc, mean, °	62.4 ± 26.0	62.6 ± 20.5	.684
Radial-ulnar deviation, mean, °	29.8 ± 10.6	45.0 ± 21.2	.405
Grip strength, mean, kg	25.3 ± 15.6	28.4 ± 6.0	.754

*Note.* 4CF = 4-corner fusion; 2CF = 2-corner fusion; 3CF = 3-corner fusion.

Of the 9 patients that underwent additional surgery, 6 patients (5 4CF and 1 2CF/3CF (*P* = .935)) developed nonunion. Of the patients who had a 4CF and nonunion, 4 had circular plate fixation and 1 had staple fixation (*P* = 1.00). Fixation type in 4CF was not associated with increased revision surgery (*P* = .80). Smoking was not associated with nonunion (*P* = .259) or revision (*P* = .223).

Six patients developed chronic pain; 2 patients with a 4CF and circular plate underwent a subsequent wrist fusion, and 1 patient with a 2CF converted to a 4CF (without resolution of pain and without further intervention). One patient with bilateral 4CFs (staple fixation) developed bilateral wrist pain; the hardware was removed from one wrist and conservative management performed with the other. One 4CF patient (staple fixation) was casted to mimic fusion. No patients were managed by chronic pain services.

### Functional Outcomes

Total wrist flexion-extension was reported for 46 wrists. Mean flexion-extension arc, 62.6 ± 20.5° in the 2CF/3CF group and 62.4 ± 26.0° in the 4CF group, was not significantly different (*P* = .983; Table 2). Radial-ulnar deviation was reported for 14 wrists; mean radial-ulnar deviation was 45 ± 21.2° for 2CF/3CF and 29.8 ± 10.6° for 4CF (*P* = .119). Grip strength was reported in 14 patients; mean grip strength was 28.5 ± 6.0 kg for 2CF/3CF and 25.3 ± 15.6 kg for 4CF (*P* = .670).

### Patient-Reported Outcomes

The survey response rate was 45% (26 (19 4CF and 7 2CF/3CF)). The mean VAS between 0 (no pain) and 10 (worst pain) was 2.9 ± 2.7 for 2CF/3CF and 3.9 ± 2.6 for 4CF (*P* = .376; Table 3). The mean Quick DASH score was 18.4 ± 9.8 for 2CF/3CF and 24.7 ± 10.7 for 4CF (*P* = .083). Twelve work modules were completed (5 2CF/3CF, 7 4CF); mean scores were 6.6 ± 2.6 for 2CF/3CF and 8.0 ± 5.2 for 4CF (*P* = .876). The sports/performing arts module was not included as only 4 were completed. Overall, 85.7% of 2CF/3CF and 57.9% of 4CF were satisfied with their surgery (*P* = .186).

**Table 3.** Results of the Patient-Reported Outcome Measures Survey After 4CF or 2CF/3CF.

Data	4CF (n = 19)	2CF and 3CF (n = 7)	*P* value
Satisfaction rate (yes/no)	57.9%	85.7%	.186
VAS, mean (0-10)	3.9 ± 2.6	2.9 ± 9.8	.597
DASH score, mean	24.7 ± 10.7	18.4 ± 9.8	.083
Work DASH score, mean	8.0 ± 5.2	6.6 ± 2.6	.876

*Note.* 4CF = 4-corner fusion; 2CF = 2-corner fusion; 3CF = 3-corner fusion; VAS = visual-analog scale; DASH = Disabilities of the Arm, Shoulder, and Hand.

## Discussion

The present study of 58 midcarpal fusions over a 10-year period demonstrates that rates of nonunion, reoperation for any cause, and progression to total wrist fusion are comparable between 4CF and 2CF/3CF when a staple fixation technique for 2CF/3CF is used. We show that wrist range of motion in flexion-extension and radial-ulnar deviation and grip strength is comparable between the traditional 4CF and 2CF/3CF procedures. The need for autologous bone grafting was significantly lower in those patients undergoing 2CF/3CF. Both 4CF and 2CF/3CF groups do not differ significantly in postoperative patient pain, satisfaction, and Quick DASH scores.

One of the proposed advantages of the 2CF, and later 3CF procedures, was the potential of increased wrist range of motion compared with a 4CF; however, to date there has not been substantial evidence to support this claim. Gaston et al^12^ found no significant improvements in flexion-extension and radio-ulnar deviation after 2CF when compared with 4CF; moreover, Gauci et al^11^ reported a greater range of motion in BA patients compared with 2CF. Consistent with their findings, our study did not observe a trend toward improved range of motion depending on the type of midcarpal fusion performed. The present study found the flexion-extension and radial-ulnar deviation arcs nearly equal between 4CF and 2CF/3CF groups.

Similar to results reported by Gaston et al^12^ comparing 2CF and 4CF, we found pain VAS scores comparable between 4CF and 2CF/3CF patients. We did observe a trend toward lower DASH scores and higher patient satisfaction in the 2CF/3CF group; however, neither of these reached statistical significance.

The lack of widespread adoption of 2CF/3CF procedures is likely attributed to high reported rates of nonunion and proximal screw migration. In the only comparative study on outcomes between 2CF and 4CF, Gaston et al^12^ found that while rates of nonunion were similar between the procedures, over 30% of patients who underwent 2CF required reoperation due to screw migration. The use of alternative fixation techniques, specifically staples, to date has only been described in a single case series, which found a decreased reoperation rate using staple fixation in 2CF/3CF.^13^ Unlike the use of screw fixation, the staple technique does not violate the remaining articular surface between the lunate and the radius. In contrast to prior work,^12^ the present study found that the overall rate of reoperation, for any cause, and rates of hardware complications were not significantly higher in the 2CF/3CF group when compared with patients undergoing 4CF. This is likely attributed to the use of staple fixation rather than compression screws in 2CF/3CF procedures. Recent studies have proposed using staple fixation for fusion to decrease the need for reoperation after 2CF.^13^ Although the sample size in this study is small, nonunion rates after 4CF do not appear to increase with the use of staples. The nonunion rate between the staple fixation and other methods of fusion does not statistically differ in our study.

Triquetral excision is routinely performed by some surgeons to reduce the risk of pisotriquetral arthritis and ulnotriquetral abutment in patients undergoing 2CF/3CF.^11,12^ While triquetral excision was not a routine component of the 2CF/3CF procedures at our institution, no patients in this group developed ulnar-abutment symptoms, pisotriquetral arthritis, or required pisiform excision.

Autologous bone grafting was used in 86% of patients who underwent a 4CF and used in only 22% of patients undergoing 2CF/3CF. By omitting the harvest and placement of bone graft, in addition to fusing fewer joints, the operative time would be expected to decrease with 2CF and 3CF, although the present study did not perform a comparison of operative times.

Our study is limited by the nature of its retrospective design. There may be baseline differences, such as disease severity, between patients selected to undergo 2CF/3CF and the traditional 4CF which could confound our results. While our surgeons generally selected a 3CF for patients with a Viegas type II lunate, over 2CF, indications for selecting 4CF vs 2CF/3CF have not been well described in the literature. As data were obtained from clinical visit notes retrospectively, documentation was sometimes incomplete, thus we were not able to include all patients for functional measurements. As our survey of patient-reported outcomes was retrospective in nature, it is subject to recall bias. As with prior studies on this topic, our study has a small sample size which likely limits our ability to demonstrate statistically significant differences between clinical and patient-reported outcomes. Due to only one 3CF procedure meeting inclusion criteria, we were unable to separate 2CF and 3CF into separate groups for comparison, which should be an aim of future work. While our cohort is among the largest comparing 4CF with 2CF or 3CF, there were still comparatively few 2CF/3CF procedures. This likely reflects the lack of adoption of these techniques due to high rates of complications previously reported, however could limit our ability to detect significant differences between groups.

Although our results show promising evidence that outcomes after limited midcarpal fusions, such as 2CF, are equivalent to 4CF, with the benefit of a decreased need for bone grafting, further prospective evidence is needed on this topic. As current literature comparing outcomes after various midcarpal fusion procedures is limited to cohort studies, in which there may be baseline differences in patients selected to undergo 4CF vs 2CF/3CF, randomized studies would provide a higher level of evidence to support the conclusions made by our group, and others. Given the infrequent nature of the limited midcarpal fusion procedures, consideration to a multicentered randomized study should be given, as outcomes such as progression to total wrist fusion and symptomatic hardware were rare in our cohort. Furthermore, larger studies which enable subgroup analysis of patients who have varying severity of baseline arthritis and comorbidities may help determine if there are specific groups of patients who would be better suited for either 4CF or 2CF/3CF specifically.

## Conclusions

This study demonstrates that limited midcarpal fusion procedures, including 2CF and 3CF, have comparable rates of revision surgery, progression to TWA, and nonunion compared with patients undergoing a 4CF for advanced midcarpal arthritis from all causes. Despite the more limited arthrodesis, 2CF and 3CF do not appear to provide an increase in range of motion. Wrist range of motion and grip strength seem to be statistically similar among patients undergoing a 2CF/3CF and 4CF. We found that patient-reported outcomes after a 2CF/3CF had comparable VAS pain scores, DASH scores, and overall satisfaction when compared with patients undergoing a 4CF. Two- and three-corner fusion procedures have a statistically significantly reduced use of autologous bone grafting thereby potentially reducing operative times.

Although previous studies report high rates of hardware complications when screw fixation is used for 2CF and 3CF procedures, the rate of revision surgery in our cohort was equal between 4CF and 2CF/3CF when staple fixation was used for the 2CF/3CF. Despite a more reduced surface area of arthrodesis and staple fixation, 2CF and 3CF were not associated with higher rates of nonunion or reoperation. Two- and three-corner fusions appear to be a feasible alternative to the traditional 4CF.
